# Comparative politics and the synthetic control method revisited: a note on Abadie et al. (2015)

**DOI:** 10.1186/s41937-017-0004-9

**Published:** 2018-05-15

**Authors:** Stefan Klößner, Ashok Kaul, Gregor Pfeifer, Manuel Schieler

**Affiliations:** 1 Statistics and Econometrics, Saarland University, Bldg. C3 1, Saarbrücken, 66123 Germany; 2 Department of Economics, Saarland University, Bldg. C3 1, Saarbrücken, 66123 Germany; 3 Department of Economics, University of Zurich, Zürichbergstrasse 14, Zurich, CH-8032 Switzerland; 4 IPE Institute for Policy Evaluation, Walther-von-Cronberg-Platz 6, Frankfurt am Main, 60594 Germany; 5 Department of Economics, University of Hohenheim, 520B, Stuttgart, 70593 Germany

**Keywords:** Synthetic control methods, Cross-validation, C23, C52

## Abstract

Recently, Abadie et al. (Am J Polit Sci 59:495–510, 2015) have expanded synthetic control methods by the so-called cross-validation technique. We find that their results are not being reproduced when alternative software packages are used or when the variables’ ordering within the dataset is changed. We show that this failure stems from the cross-validation technique relying on non-uniquely defined predictor weights. While the amount of the resulting ambiguity is negligible for the main application of Abadie et al. (Am J Polit Sci 59:495–510, 2015), we find it to be substantial for several of their robustness analyses. Applying well-defined, standard synthetic control methods reveals that the authors’ results are particularly driven by a specific control country, the USA.

## Background

As a tool for policy evaluation, Abadie and Gardeazabal (2003) have introduced so-called synthetic control methods (SCM). For estimating the development of the treated unit in absence of the treatment, the basic idea of SCM is to find suitable donor weights which describe how the treated unit is synthesized by a weighted mix of unaffected control units. In this context, “suitable” means that treated and synthetic unit should resemble each other as closely as possible prior to the treatment, both with respect to the outcome of interest and with respect to so-called economic predictors. The latter are variables of predictive power for explaining the outcome. The data-driven SCM approach searches for optimal predictor weights in order to grant more importance to economic predictors with better predictive power. Properties of the SCM estimator, like (asymptotic) unbiasedness, have been developed by Abadie et al. (2010), while Gardeazabal and Vega-Bayo (2017) find that the SCM estimator performs well as compared to alternative panel approaches.

Over the last few years, many studies have applied SCM across several fields, e.g., Acemoglu et al. (2016) (political connections), Cavallo et al. (2013) (natural disasters), Gobillon and Magnac (2016) (enterprise zones), or Kleven et al. (2013) (taxation of athletes). Recently, the SCM approach has been expanded by Abadie et al. (2015) (German reunification) to incorporate *cross-validation*: the predictor weights, whose data in the training period (first part of the pre-treatment period) are used to find optimal donor weights for synthesizing the treated unit, are selected such that the out-of-sample error in the validation period (second part of the pre-treatment period) is minimized.

When measuring the effect of the 1990 reunification on Germany’s GDP per capita using the software package R, Abadie et al. (2015) found the following predictor weights: 44.2% (GDP per capita), 24.5% (investment rate), 13.4% (trade openness), 10.7% (amount of schooling), 7.2% (inflation rate), and 0.1% (industry share of value added). These predictor weights led to Germany being synthesized by Austria (42%), the United States (22%), Japan (16%), Switzerland (11%), and the Netherlands (9%).

When trying to replicate these results using the software package Stata, however, we found different predictor weights: 84.5% (GDP), 4.5% (investment), 5.1% (trade), 4.2% (schooling), 0.5% (inflation), and 1.2% (industry). The corresponding synthetic Germany was slightly different from the one obtained by Abadie et al. (2015): it consisted of Austria (43%), the USA (22%), Japan (15%), Switzerland (11%), and the Netherlands (9%)^1^. We had sorted the countries alphabetically, while Abadie et al. (2015) had used a different ordering^2^. Although, in theory, the ordering should have no effect on the estimation results (neither should the respective software package), we recalculated all weights using the ordering that had been used by Abadie et al. (2015). Surprisingly, we got yet *another* set of predictor weights: 71.0% (GDP), 11.1% (investment), 7.9% (trade), 6.4% (schooling), 2.7% (inflation), and 0.9% (industry). The corresponding weights for the countries synthesizing Germany were much closer to, but still different from the values found by Abadie et al. (2015)^3^.

Closer inspection shows that the failure to reproduce the results of Abadie et al. (2015) is not due to software problems, but stems from the newly introduced cross-validation technique. In fact, all the above mentioned predictor weights deliver identical values for the cross-validation criterion, thus they are all equivalent solutions of the cross-validation approach. Hence, the cross-validation technique is (in most applications) not well-defined, since the predictor weights are not uniquely defined. As the cross-validation technique allows many different equivalent predictor weights, the results obtained by Abadie et al. (2015) are arbitrary in the sense that the authors could have obtained different results if they had used other software or organized the data differently.

We therefore investigate the corresponding ambiguity by conducting large-scaled Monte Carlo studies. The variation of the estimated post-treatment development of West German GDP is very small, with all estimates being significantly above Germany’s actual GDP. Concerning several robustness studies of Abadie et al. (2015), however, we find quite large amounts of ambiguity, in particular for the so-called in-space placebo and leave-one-out studies. Developing a rule of thumb, we can show that the amount of ambiguity depends on the difference between the number of predictors and the number of donor units that obtain positive weights in the training period. In most applications, this difference is positive. Thus, using the cross-validation cannot be recommended and standard synthetic control methods should be applied instead. When doing so, we confirm the main result of Abadie et al. (2015), indicating a significant drop in West German GDP due to the reunification. In contrast to Abadie et al. (2015), however, detecting such a significant gap crucially hinges on including US data.

The remainder of the paper unfolds as follows: the “Methods” section describes the synthetic control method with and without cross-validation and elaborates on the reasons why the cross-validation technique is typically not well-defined, while the standard SCM approach does not suffer from this problem. We then analyze the extent to which the results of Abadie et al. (2015) are prone to ambiguity and compare them to those under the standard synthetic control approach. The “Conclusions” section ends the paper.

## Methods

### Synthetic control methods

In the following, we describe how synthetic control methods work both with and without the cross-validation technique. Many additional explanations, in particular on how to select potential comparison units and predictor variables, are provided in Abadie et al. (2015)^4^.

**Fig. 1:**
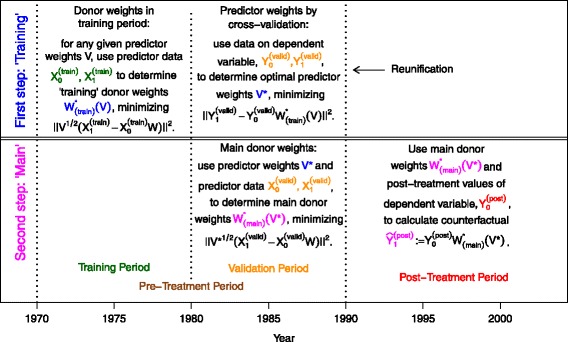
Schematic overview for the cross-validation technique in SCM. Notes: predictor and donor weights have been named and color-coded according to the steps during which they are computed, while the data have been named and color-coded according to the different periods they belong to

For the synthetic control method, we have two types of data: the variable of interest, often denoted by the letter *Y*, and predictor variables, usually denoted by *X*. These are considered both for a unit that has at some point in time been “treated,” usually denoted by the subscript “_1_,” and for so-called donor units. The latter are units not too different from the first one, but unaffected from the treatment, and denoted by the subscript “_0_.” In the example discussed throughout this paper, the treated unit is Germany which has been reunified in 1990, the variable of interest is GDP per capita, and predictors are (pre-treatment) GDP per capita, a measure for trade openness, the inflation rate, the industry share of value added, the amount of schooling attained, and the investment rate. The donor units consist of sixteen OECD countries^5^ for which the synthetic control method determines non-negative so-called donor weights *W*: these weights describe to what extent each donor country is used to produce a “synthetic” (i.e., counterfactual) Germany. Thereby, the weights should be such that synthetic Germany mimics actual Germany as well as possible with respect to the (pre-treatment) predictor variables. For the example at hand, this means that the differences between actual and synthetic Germany with respect to GDP per capita, trade openness, inflation rate, industry share, schooling, and investment rate should be as small as possible. As we have six predictors (*k*=6), operationalizing the last statement requires introducing some weighting scheme. These non-negative so-called predictor weights are usually denoted by *v*_*m*_ or *V*, and the cross-validation technique introduced in Abadie et al. (2015) is a new method to determine such weights. To this end, the pre-treatment period is divided into two parts, a training and a validation period. For the case of the German reunification, the training period is 1971–1980, while the validation period is 1981–1990, see Fig. 1 for a schematic overview of how the cross-validation approach is defined.

In the training period, one makes use of the (*k*×*J*)-matrix $X_{0}^{(\text {train})}$ and the *k*-dimensional vector $X_{1}^{(\text {train})}$, containing time averages of the predictors’ data for the donor units and the treated unit, respectively^6^. For any predictor weights *V*=(*v*_1_,…,*v*_*k*_), the donor weights $W^{*}_{(\text {train})}(V)$ in the training period are defined as the minimizer of $\sum \limits _{m=1}^{k} v_{m} \left (X_{1m}^{(\text {train})}-X_{0m}^{(\text {train})}W\right)^{2}$ with respect to *J*-dimensional non-negative donor weights *W* summing to unity, i.e., as the solution of 
1

where  denotes the vector of ones, while $X^{(\text {train})}_{1m}$ and $X^{(\text {train})}_{0m}$ denote the *m*-th component and row of $X^{(\text {train})}_{1}$ and $X^{(\text {train})}_{0}$, respectively.

In the validation period, one uses the (*L*×*J*)-matrix $Y_{0}^{(\text {valid})}$ and the *L*-dimensional vector $Y_{1}^{(\text {valid})}$, containing the variable of interest’s data for the validation period^7^. The cross-validation defines predictor weights $V^{*}=\left (v_{1}^{*},\hdots,v_{k}^{*}\right)$ as those weights that minimize the out-of-sample error $\left |\left | Y_{1}^{(\text {valid})}-Y_{0}^{(\text {valid})}W^{*}_{(\text {train})}(V) \right |\right |^{2}$ over *V*, i.e., *V*^∗^ is a minimizer of^8^2

where we have normalized the predictor weights to sum to unity^9^.

These predictor weights *V*^∗^ are then used to determine $W^{*}_{(\text {main})}$ as the minimizer of 
3

where the (*k*×*J*)-matrix $X_{0}^{(\text {valid})}$ and the *k*-dimensional vector $X_{1}^{(\text {valid})}$ contain time averages of the predictors’ data for the validation period, which for the application at hand ranges from 1981 to 1990.

Thus, the synthetic control method with cross-validation is a two-step procedure. First, in the so-called “training” step, *V*^∗^ is determined by minimizing the cross-validation criterion, thereby making use of “training” weights $W^{*}_{(\text {train})}(V)$ as defined by Eq. (1). Then, in the second, so-called “main” step, these predictor weights *V*^∗^ are used to determine the “main” donor weights $W^{*}_{(\text {main})}(V^{*})$ by Eq. (3). These “main” donor weights $W^{*}_{(\text {main})}(V^{*})$ are then employed for synthesizing the treated unit. Again, this is visualized in Fig. 1.

In contrast, the standard synthetic control method consists of only one step, not distinguishing between a training and validation period.

Instead, all pre-treatment data are used, in our application those from 1971 to 1990, to build the following quantities: the (*k*×*J*)-matrix $X_{0}^{(\text {pre})}$ and the *k*-dimensional vector $X_{1}^{(\text {pre})}$, containing time averages of the predictors’ data for the donor units and the treated unit, respectively, as well as the $(\tilde L\times J)$-matrix $Y_{0}^{(\text {pre})}$ and the $\tilde L$-dimensional vector $Y_{1}^{(\text {pre})}$, containing the variable of interest’s pre-treatment data for the donor units and the treated unit, respectively^10^. For given predictor weights *V*=(*v*_1_,…,*v*_*k*_), the standard SCM approach defines the donor weights *W*^∗^(*V*) as the minimizer of $\sum \limits _{m=1}^{k} v_{m} \left (X_{1m}^{(\text {pre})}-X_{0m}^{(\text {pre})}W\right)^{2}$, i.e., as the solution of 
4

Optimal predictor weights *V*^∗^ are then determined by minimizing the in-sample error^11^, i.e., as a solution of 
5

**Fig. 2:**
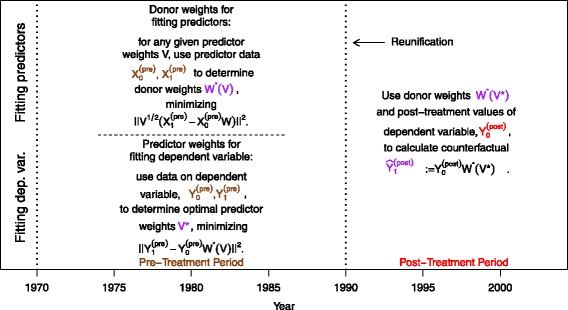
Schematic overview for the “standard” SCM technique. Notes: data have been named and color-coded according to the different periods they belong to

The donor weights *W*^∗^(*V*^∗^) are then used for synthesizing the treated unit. For a schematic overview of standard SCM, see Fig. 2.

### Well-definedness of synthetic control methods

A crucial insight as to why the cross-validation technique of Abadie et al. (2015) is not well-defined is the fact that, typically, there is no *unique* minimizer of the out-of-sample error $\left |\left | Y_{1}^{(\text {valid})}\,-\,Y_{0}^{(\text {valid})}W^{*}_{(\text {train})}(V)\right |\right |^{2}$. Thus, Eq. (2) does not define *V*^∗^ unambiguously. The reason is that the mapping $W^{*}_{(\text {train})}$ defined by Eq. (1) is often not injective—it regularly happens that $W^{*}_{(\text {train})}(\widetilde V)$ and $W^{*}_{(\text {train})}(V)$ coincide although $\widetilde V$ and *V* are different after scaling. Less formally, it is often the case that different predictor weights lead to the same “training” weights. The problem of the cross-validation approach is that such different predictor weights $\widetilde V$ and *V*, although scaled and entailing identical $W^{*}_{(\text {train})}(\widetilde V)=W^{*}_{(\text {train})}(V)$, typically lead to different $W^{*}_{(\text {main})}(\widetilde V)\neq W^{*}_{(\text {main})}(V)$ in the main step of Eq. (3).

Actually, this is the reason behind the diverging results described above: all predictor weights given earlier, those found by Abadie et al. (2015) as well as our results obtained using Stata with two different orderings for the donor countries, are equivalent solutions of Eq. (2). This can be seen from the “*W* weights training” rows of columns “ADH,” “Orig.,” and “Alph.” of Table 1 below. All these different predictor weights produce the same $W^{*}_{(\text {train})}$ in Eq. (1), leading to an identical out-of-sample error of 67.7. However, although these different predictor weights *V* are equivalent with respect to Eq. (1), i.e., produce the same donor weights $W^{*}_{(\text {train})}$ and therefore the same synthetic Germany in the training period, they are *not* equivalent with respect to Eq. (3). More specifically, the corresponding donor weights $W^{*}_{(\text {main})}$ for the main application do not coincide (cf. the “*W* weights main” rows of columns “ADH,” “Orig,” and “Alph.” of Table 1). Overall, hence, one obtains different synthetic versions for the treated unit, leading to different estimates for the post-treatment development of the treated unit in absence of the intervention, and potentially to diverging conclusions about the effect of the intervention. Thus, in the end, the cross-validation technique introduced by Abadie et al. (2015) is not properly defined, typically leading to ambiguous estimates of the treatment effect.

While $W^{*}_{(\text {train})}$*in general* is not injective, it depends on the respective application whether or not there exist several different predictor weights minimizing the out-of-sample error. In some applications, there might be an up to scaling unique minimizer, making the cross-validation technique well-defined, while in other applications, there might exist many different minimizers. In the latter case, it is not clear how large the set of these minimizers will be. In the Appendix, we therefore elaborate on a heuristic rule of thumb that allows to get an idea about the amount of ambiguity. It turns out that the decisive quantity in this context is the difference *k*−*α* between the number of predictors used, *k*, and the number of donor units that obtain positive weights in the training period, $\alpha :=\#\left \{j:W^{*}_{(\text {train}),j}>0\right \}$. If the difference *k*−*α* is positive, the predictor weights will typically not be uniquely defined by the cross-validation technique, with a generically increasing amount of ambiguity the larger *k*−*α*. In case of the German reunification, six predictors (GDP, trade openness, inflation, industry share, schooling, and investment rate) are used, but only five donor units obtain positive weights in the training period (the USA, Austria, Switzerland, Japan, and Australia, cf. Table 1), thus *k*−*α*=6−5=1>0. Consequently, there exist many solutions for determining the predictor weights by the cross-validation technique, but the amount of ambiguity is expected to be rather small.

Eventually, the standard synthetic control method does *not* suffer from similar problems. In a nutshell, the reason is that the standard SCM method consists of only one step, while the cross-validation technique is a two-step procedure. When using the cross-validation technique, the non-uniqueness of the predictor weights entails ambiguous donor weights in the second, main step of the cross-validation technique. In this case, the uniquely defined donor weights in the first, training step, are of no help. For the standard method, there are generically many different solutions *V*^∗^ to Eq. (5), in complete analogy to the cross-validation method and Eq. (2). Again, the reason is that the mapping *W*^∗^ defined by Eq. (4) is often not injective: it regularly happens that $W^{*}(\widetilde V)$ equals *W*^∗^(*V*) although $\widetilde V$ and *V* are different after scaling. However, in contrast to the cross-validation method, this is not a problem, as predictor weights are not used as input for a second step with different predictor data. Instead, the *unique* donor weights *W*^∗^(*V*^∗^) are the only quantity needed to synthesize the treated unit and estimate treatment effects, and therefore the standard synthetic control method leads to well-defined estimators, in contrast to the cross-validation approach. This can also be seen from Table 3 below, where different predictor weights obtained by different software and different settings all entail identical donor weights.

## Results and Discussion

### Ambiguity of results using the cross-validation technique

For the upcoming analysis, we retrieved from the AJPS Data Archive on Dataverse (https://dataverse.harvard.edu/dataverse/ajps) both the data and all code of Abadie et al. (2015).

We also followed Abadie et al. (2015) by using R (R Core Team 2014) in combination with package Synth (Abadie et al. 2011). In particular, we first ran the code supplied by Abadie et al. (2015), storing all the results, especially the results for the donor units’ weights in the training period. We then conducted large-scale Monte Carlo studies, searching for predictor weights that also lead to these donor weights in the training period, i.e., “training-equivalent” predictor weights which also minimize Eq. (2). These were then used to calculate the corresponding donor weights for the main period, GDP estimates, and follow-up quantities^12^.

Table 1 summarizes the results for the predictor weights *V* and donor weights *W*: columns “ADH,” “Orig.,” and “Alph.” contain the results obtained by Abadie et al. (2015), by Stata using the same ordering of donor countries as did Abadie et al. (2015), and by Stata using the donor countries in alphabetical order, respectively. Columns “Min.” and “Max.” contain the smallest and largest values obtained in our Monte Carlo study, respectively. We find the weights of some predictors to vary substantially. For instance, the *V* weight of GDP can take values between 38.5 and 87.7%, while the inflation rate may be almost irrelevant with a weight of nearly zero but may also be taken into considerable account when its weight is 8.1%. For the composition of synthetic Germany, we find similar ambiguity: the weight of Austria varies between 41.6 and 47.1%, the Netherlands can be essentially unimportant with a weight of only 0.6% but also contribute 9.4% to synthetic Germany. In some cases, Germany is even synthesized by six instead of five countries, when the UK or Norway are attributed small but positive weights, respectively.

**Fig. 3:**
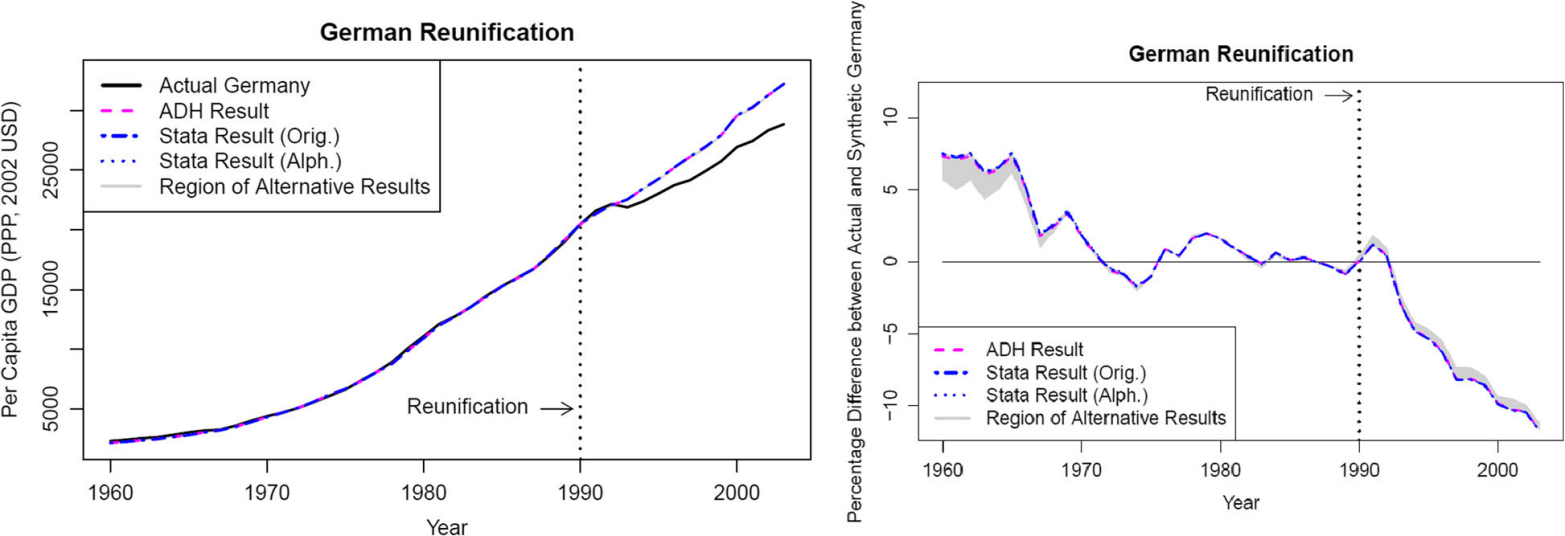
GDP timelines of actual Germany and various versions of synthetic Germany. Notes: left part: purchasing power parity (PPP)-adjusted GDP in 2002 US dollars, right part: percentage differences between actual Germany and synthetic Germany. “ADH” denotes the results obtained by Abadie et al. (2015), “Stata Result (Orig.)” and “Stata Result (Alph.)” stand for the results we obtained using Stata, using the same ordering of the donors as did Abadie et al. (2015) (“Orig.”), and alphabetical ordering (“Alph.”), respectively

Figure 3 (the left part of which corresponds to Fig. 3 of Abadie et al. (2015)) shows the timelines of GDP for actual Germany as well as several versions of synthetic Germany. From the original timelines, differences between the various versions of synthetic Germany are barely visible. Closer inspection shows that the range due to ambiguous weights varies between approximately 5.11 and 228.90 US dollars per capita, on average taking a value of 72.57 dollars. As German GDP per capita rose from roughly 3,000 US dollars in 1960 to almost 30,000 US dollars in 2003, we accompany these figures and timelines by what might be called a “relative gap plot,” namely the percentage difference between actual and synthetic Germany. The gray area, which displays the range of ambiguity due to different but equivalent results, now becomes visible. Overall, however, it is quite small, taking values between 0.06 and 2.29%, with an average relative difference of 0.64%. Again, this indicates that although donor weights are ambiguous, the conclusion with respect to a gap in German GDP after the reunification remains valid.

We now turn our attention to the in-space placebo study which artificially reassigns the reunification to all donor countries, thus treating Germany as a donor country, while at the same time, one of the donor countries takes the role of the treated unit. To evaluate the results, one calculates the ratios of post-treatment differences between actual and synthetic GDP values over corresponding pre-treatment differences. The results are displayed in Fig. 4 which, as a special case, contains Fig. 5 of Abadie et al. (2015). We find rather large ranges of ratios for some countries (Germany, Norway, the USA, Spain, Switzerland, the UK, and the Netherlands), and small to (almost) no ranges of ratios for other countries^13^. In line with our heuristic rule of thumb, the countries with large ranges are characterized by rather small numbers of donor countries contributing in the training period: Switzerland (one donor country), the USA, Portugal, Spain (two donor countries), the UK, the Netherlands, Japan (three donor countries), and Norway (four donor countries). The range of ratios is largest for Norway, with a value of 3.28, the average range size is 0.87. Overall, notwithstanding the significant ambiguity of these ratios, the ratio for Germany is by far the largest, indicating that the reunification had a significant impact on German GDP per capita.

Table 2 and Fig. 5 (the left part of which corresponds to Fig. 6 of Abadie et al. (2015)) show the results for the case when the U.S. data is removed from the sample—a so-called “leave-one-out” analysis which, in the original Abadie et al. (2015) study, backs up the main finding. Here, the ambiguity with respect to the predictor weights $W_{(\text {main})}^{*}$ is quite pronounced. For instance, Austria may be used for synthesizing Germany with a weight of up to 67% but may also be completely neglected for synthesizing, as in the solution found by Abadie et al. (2015). On the other hand, the country obtaining the largest weight in the solution of Abadie et al. (2015), Switzerland, may not be used at all for synthesizing Germany. This rather large ambiguity is again in line with our heuristic rule of thumb, as in this case, there are only four countries obtaining positive training weights $W_{(\text {train})}^{*}$, see Table 2. Correspondingly, the gray area in Fig. 5 indicating the range of ambiguity now is very large^14^ and the original result found by Abadie et al. (2015) is extreme under all equivalent results: all other possible results show smaller post-treatment gaps between actual and synthetic Germany’s GDP per capita, raising the question whether the gap in GDP due to the reunification crucially hinges on the U.S. acting as a donor country synthesizing Germany. The relative gap plot of Fig. 5 also shows that the gap of approximately 7% in 2003 is not larger than the pre-treatment approximation error of about 7% in the early 1970s, strengthening the doubts whether there is still a significant gap in German GDP per capita after the reunification when the US data is removed. Correspondingly, the ratio of post-treatment over pre-treatment differences is only 4.64, quite a small value as compared to the ratios of the in-space placebo study. Therefore, in contrast to what Abadie et al. (2015) find, it seems that including the US data is essential for obtaining a significant gap in GDP between actual and synthetic Germany.

### Results using standard SCM

As a well-defined alternative to applying the cross-validation technique, we will now use the standard synthetic control method to analyze the reunification’s effect on West Germany’s GDP. Applying this method, we find Germany to be synthesized by Austria, the USA, Switzerland, Japan, and the Netherlands, see Table 3. We found identical donor weights when using R, Stata with the original ordering, and Stata with alphabetical ordering of donor countries, in line with the standard SCM technique being well-defined. Furthermore, the table also shows that, as discussed above, these donor weights can be obtained by completely different predictor weights.

**Fig. 6:**
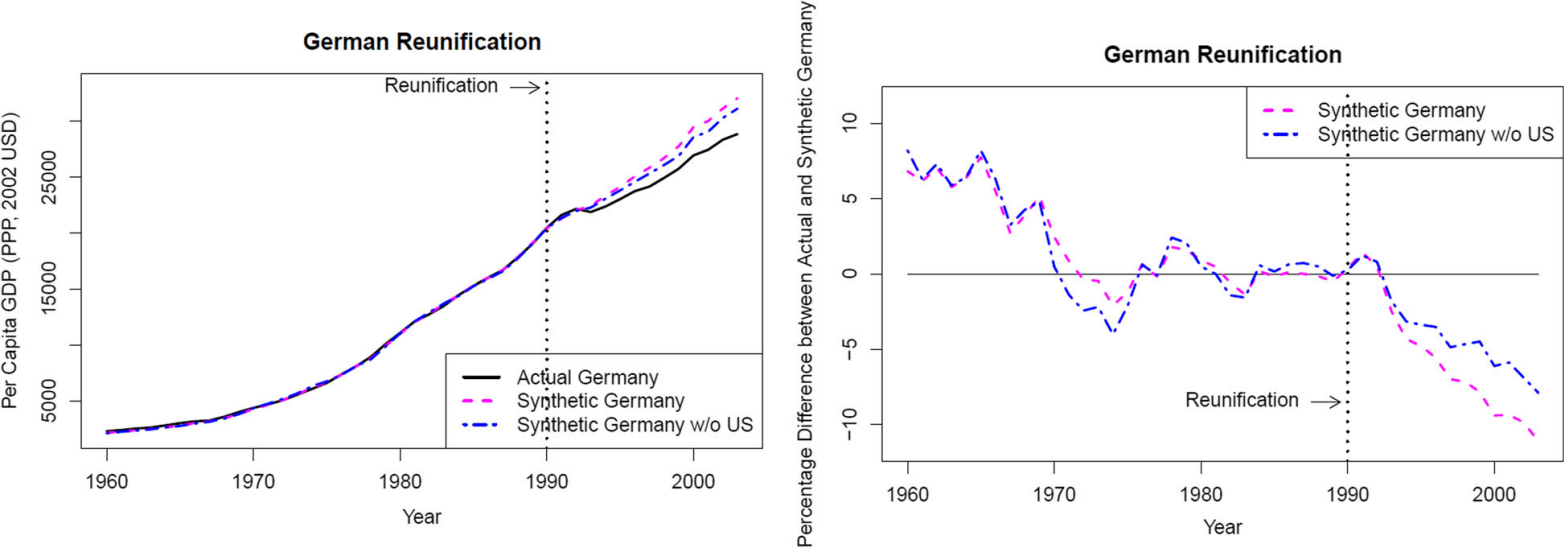
GDP timelines of actual Germany and two versions of synthetic Germany. Notes: left part: purchasing power parity (PPP)-adjusted GDP in 2002 US dollars; right part: percentage differences between actual Germany and synthetic Germany. “Synthetic Germany” stands for the results obtained when using standard SCM, “Synthetic Germany w/o US” is the corresponding result when the US data is removed from the sample

**Fig. 7:**
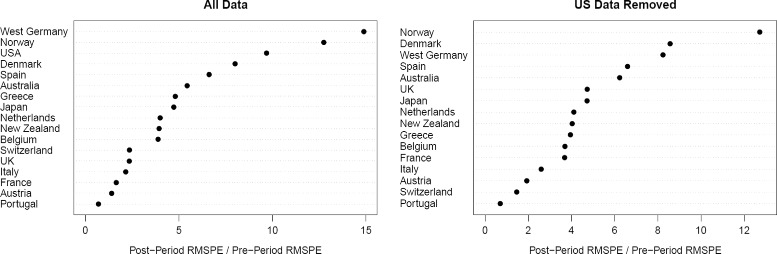
Ratios of post-treatment over pre-treatment root mean square prediction error (RMSPE) for in-space placebos, using standard SCM. Notes: left part: results when using all data; right part: results after removing US data from the sample

The corresponding timelines for GDP per capita are displayed in Fig. 6, the results for the in-space placebo study can be found in the left part of Fig. 7. These results are very similar to those obtained when using the cross-validation technique: after the reunification, Germany suffered from a significant loss in GDP per capita which amounted to roughly 11% in 2003.

**Table 3:** Results from standard SCM (predictor weights *V*, donor weights *W*) obtained in different ways

	R	Stata Orig.	Stata Alph.
GDP per capita	48.5	62.9	0.0
Trade openness	0.0	0.0	0.0
Inflation rate	0.1	0.0	0.0
Industry share	0.0	0.0	0.0
Schooling	30.5	17.3	92.0
Investement rate	20.9	19.8	8.0
USA	16.0	16.0	16.0
Austria	62.6	62.6	62.6
Netherlands	1.5	1.5	1.5
Switzerland	13.1	13.1	13.1
Japan	6.8	6.8	6.8

Notes: “R” stands for results obtained using R, “Stata Orig.” are results from Stata with the same ordering of donors as in the code of Abadie et al. (2015), “Stata Alph.” denotes results from Stata with donors sorted alphabetically. All numbers are given in percent, suppressing donors with weight less than 1%

Figure 6 as well as the right part of Fig. 7 show the results after removing the US data from the sample. The gap between actual and synthetic Germany reduces to approximately 8%, and the ratio of post-treatment differences to pre-treatment differences shrinks from 14.9 to 8.2, which is much smaller than the ratio for Norway (12.7). Therefore, also when using the standard SCM approach, the US data is essential for detecting a significant gap in German GDP per capita caused by the reunification.

## Conclusions

The synthetic control method is an important tool in policy evaluation which has been expanded by Abadie et al. (2015), who introduce the cross-validation technique for selecting predictor weights. In this paper, we have shown that this technique is not well-defined because it hinges on predictor weights which in many applications will not be uniquely defined. When using synthetic control methods in combination with cross-validation, one might therefore arrive at ambiguous results and conclusions.

As far as theory is concerned, we derive a heuristic rule of thumb which relates non-uniqueness of the predictor weights to the difference between the number of predictors and the number of donor units that synthesize the unit of interest in the training period. If this difference is positive, which is the case in most applications, predictor weights based on cross-validation are typically not uniquely defined, and the ambiguity with respect to this non-uniqueness usually becomes larger the more this difference increases.

Empirically, examining the German reunification using the data of Abadie et al. (2015), we find that the amount of ambiguity is rather small as far as the main application is concerned. With respect to several robustness studies, however, the ambiguity implied by the predictors’ non-uniqueness is significant, in particular for the leave-one-out and in-space placebo studies.

The failure of synthetic control methods with cross-validation is no failure of synthetic control methods as such. One can simply stick to the standard synthetic control method without cross-validation since it does not contain a second estimation step for which the predictor weights’ uniqueness is crucial. When doing so for the example of the German reunification, we mostly confirm the results of Abadie et al. (2015)—there is a significant gap in German GDP due to the reunification. With respect to robustness, however, we find, in contrast to Abadie et al. (2015), that this result crucially depends on the US data being included in the estimation. After removing the US data from the sample, the estimated gap in GDP after the German reunification becomes much smaller and is no longer significant according to the in-space placebo study.

## Appendix

## Theory on predictor weights by cross-validation

Let *V*^∗^ be a solution to Eq. (2) and $W^{*}_{(\text {train})}(V^{*})$ be the corresponding minimizer of Eq. (1). We denote by  the set of all scaled predictor weights *V* that lead to the same “training” weights as *V*^∗^.

The cross-validation technique is typically not well-defined if predictor weights $\widetilde V\in {\mathcal {V}}$ exist that are different from *V*^∗^. Then, $\widetilde V$ is also an optimizer of the out-of-sample error, but leading to “main” weights $W^{*}_{(\text {main})}(\widetilde V)$ which typically do not coincide with the corresponding “main” weights belonging to *V*^∗^^15^: $W^{*}_{(\text {main})}(\widetilde V)\neq W^{*}_{(\text {main})}(V^{*})$. Thus, well-definedness of the cross-validation technique crucially hinges on ${\mathcal {V}}$ being a singleton. Furthermore, the larger ${\mathcal {V}}$, the more different weights for synthesizing, $W^{*}_{(\text {main})}(\widetilde V)$ for $\widetilde V\in {\mathcal {V}}$, will typically exist, and the larger the amount of ambiguity of the cross-validation approach usually will be.

To develop a rule of thumb which sheds some light on how large ${\mathcal {V}}$ and thus the resulting ambiguity of the cross-validation technique are, we state the following Lemma.

### **Lemma 1**

For any given predictor weights *V*, an optimizer *W*^∗^ of Eq. (1) must fulfill the following conditions^16^:

1. for all *j* running through the components of *W*^∗^, with *e*_*j*_ denoting the *j*-th unit vector:
  6 $$ \begin{aligned} d_{j}(W^{*},V):=&\sum\limits_{m=1}^{k} v_{m} \left(X_{1m}^{(\text{train})}-X_{0m}^{(\text{train})}W^{*}\right) \\&\times X_{0m}^{(\text{train})} (W^{*}-e_{j})\geq 0, \end{aligned}  $$
2. *d*_*j*_(*W*^∗^,*V*)=0 for all *j* with $W^{*}_{j}>0$.

### *Proof*

For every *j*, consider

$${} f_{j}(\delta):=\sum\limits_{m=1}^{k} v_{m} \left(X_{1m}^{(\text{train})}- X_{0m}^{(\text{train})} \left((1-\delta)W^{*}+\delta e_{j} \right) \right)^{2}. $$

The derivative of *f*_*j*_ at *δ*=0,

$${{} \begin{aligned} f_{j}^{\prime}(0)&= 2\sum\limits_{m=1}^{k} v_{m} \left(X_{1m}^{(\text{train})}-X_{0m}^{(\text{train})}W^{*}\right) X_{0m}^{(\text{train})} (W^{*}-e_{j})\\&= 2 \, d_{j}(W^{*},V), \end{aligned}} $$

must be non-negative, as otherwise the convex combination (1−*δ*)*W*^∗^+*δe*_*j*_ would for small positive *δ* yield a smaller value in (1) than does *W*^∗^. For *j* with $W^{*}_{j}>0$, the vector (1−*δ*)*W*^∗^+*δe*_*j*_ will have non-negative components summing to unity even for negative *δ* that are small enough in absolute value. Therefore, $f_{j}^{\prime }(0)$ must vanish in that case, as otherwise (1−*δ*)*W*^∗^+*δe*_*j*_ for small negative *δ* would yield a smaller value than *W*^∗^ in Eq. (1). □

Fixing $W^{*}:=W^{*}_{(\text {train})}(V^{*})$, Lemma 1 states the conditions *V* must fulfill to belong to ${\mathcal {V}}$: *d*_*j*_(*W*^∗^,*V*)≥0 for all *j* with $W^{*}_{j}=0$, and *d*_*j*_(*W*^∗^,*V*)=0 for all *j* with $W^{*}_{j}>0$. As *d*_*j*_(*W*^∗^,*V*) is a linear function of *v*_1_,…,*v*_*k*_, the conditions for *V* to belong to ${\mathcal {V}}$ thus consist of linear equations and inequalities: for the *k* unknown quantities *v*_1_,…,*v*_*k*_, we have *α* linear equations and *J*−*α* linear inequalities, with *J* denoting the number of donor units, and $\alpha :=\{j:W^{*}_{j}>0\}$, the number of donor units which obtain positive weights in the “training” period. As a rule of thumb, we thus have the following^17^:

### **Rule of Thumb 1**

The cross-validation method is typically not well-defined if the difference *k*−*α* between the number of economic predictors (*k*) and the number of donor units with positive *W* weight in the “training” period (*α*) is positive. The larger the difference *k*−*α*, the larger is typically the ambiguity induced by the cross-validation technique.

Finally, notice that ${\mathcal {V}}$ is a convex set, as the conditions in Lemma 1 are linear in the *V* weights. In particular, this entails that as soon as ${\mathcal {V}}$ is not a singleton, ${\mathcal {V}}$ contains infinitely many elements, with its dimension typically increasing with *k*−*α*.

## Footnotes

1 The seemingly small differences of the countries’ weights delivered by Abadie et al. (2015) (also called ADH subsequently) and Stata are actually more pronounced, cf. the “*W* Weights Main” entries of columns “ADH” and “Alph.” in Table 1 below.

**Table 1:** Results (predictor weights *V*, donor weights *W* for main application and in training period, cross-validation criterion) obtained in different ways

		ADH	Orig.	Alph.	Min.	Max.
*V* weights	GDP per capita	44.2	71.0	84.5	38.5	87.7
	Trade openness	13.4	7.9	5.1	4.4	14.6
	Inflation rate	7.2	2.7	0.5	0.0	8.1
	Industry share	0.1	0.9	1.2	0.0	1.3
	Schooling	10.7	6.4	4.2	3.7	11.6
	Investment rate	24.5	11.1	4.5	2.9	27.2
*W* weights main	USA	21.9	22.1	22.0	20.1	22.1
	UK	0.1	0.0	0.0	0.0	1.8
	Austria	41.8	42.2	43.1	41.6	47.1
	Netherlands	9.0	9.2	8.5	0.6	9.4
	Norway	0.1	0.0	0.0	0.0	1.9
	Switzerland	11.1	10.9	10.8	10.8	13.7
	Japan	15.5	15.7	15.4	9.9	15.7
*W* weights training	USA	13.5	13.5	13.5	13.5	13.5
	Austria	50.7	50.7	50.7	50.7	50.7
	Switzerland	16.6	16.6	16.6	16.6	16.6
	Japan	14.6	14.6	14.6	14.6	14.6
	Australia	4.5	4.5	4.5	4.5	4.5
C-V criterion	RMSPE	67.7	67.7	67.7	67.7	67.7

Notes: “ADH” stands for the results of Abadie et al. (2015), “Orig.” are results from Stata with the same ordering of donors as in the code of Abadie et al. (2015), “Alph.” denotes results from Stata with donors sorted alphabetically, “Min.” and “Max.” denote minimal and maximal values, respectively, found under the condition that the corresponding predictor weights *V* lead to identical donor weights *W* in the training period. All numbers are given in percent, suppressing donors with weight less than 1%

2 The data file used by (Abadie et al. 2015) implicitly orders the countries by the U.S., the UK, Austria, Belgium, Denmark, France, West Germany, Italy, the Netherlands, Norway, Switzerland, Japan, Greece, Portugal, Spain, Australia, New Zealand.

3 See the “*W* Weights Main” entries of columns “ADH” and “Orig.” in Table 1 below.

4 As we need a very formal representation of the SCM techniques for later use, we provide a rather mathematical translation of the verbal description and R-code given by Abadie et al. (2015).

5 For more details on variables as well as donor choice, see Abadie et al. (2015, p. 509).

6 *J* denotes the number of donor units used to synthesize the treated unit, for the application under consideration, the German reunification, the *J*=16 donor units are given by the U.S., the UK, Austria, Belgium, Denmark, France, Italy, the Netherlands, Norway, Switzerland, Japan, Greece, Portugal, Spain, Australia, and New Zealand.

7 For the application at hand, annual data from 1981 to 1990 are used, resulting in *L*=10.

8 Abadie et al. (2015, p. 502): “Intuitively, the cross-validation technique selects the weights *v*_*m*_ that minimize out-of-sample prediction errors.”

9 As $W^{*}_{(\text {train})}(\alpha V)=W^{*}_{(\text {train})}(V)$ for all predictor weights *V* and positive constants *α*>0, one may assume without loss of generality that predictor weights are always scaled such that their components sum to unity, see, e.g., (Abadie and Gardeazabal 2003, p. 128), (Abadie et al. 2015, Footnote 5, p. 497)

10 For the German reunification, annual data for the pre-treatment time span 1971–1990 are used, resulting in $\tilde L=20$.

11 There exists another method to determine predictor weights, the so-called regression-based method, which however is rarely used in practice.

12 More precisely, we simulated values for *v*_1_,…,*v*_*k*_ by independent draws from the Cauchy distribution, solved Eq. (1), and checked whether $W^{*}_{(\text {train})}(v_{1},\hdots,v_{k})$ was up to four digits equal to the “training” weights given in Table 1. If this was the case, we computed the corresponding “main” *W* weights and the other follow-up quantities like GDP estimates, etc., and stored the corresponding *V* weights for later use. The whole procedure was repeated for several billion draws of the *V* weights.

13 Note that ranges might be underestimated as these were calculated from the extensive, yet limited Monte Carlo study that we conducted.

14 In terms of U.S. dollars, ranges in GDP per capita vary between 681.8 and 1,979, with an average range of 1,198 dollars. In terms of relative differences between actual and counterfactual GDP per capita, the smallest and largest ranges are 2.48 and 33.15 percent, respectively, while the average range is 14.97 percent.

**Table 2:** Results (predictor weights *V*, donor weights *W* for main application and in training period, cross-validation criterion) obtained in different ways

		ADH	Min.	Max.
*V* weights	GDP per capita	23.0	0.0	23.0
	Trade openness	48.5	48.5	63.1
	Inflation rate	22.1	22.1	28.9
	Industry share	0.0	0.0	1.3
	Schooling	0.0	0.0	12.9
	Investment rate	6.5	1.3	9.2
*W* weights main	UK	22.1	0.0	22.2
	Austria	0.0	0.0	67.0
	Denmark	0.0	0.0	17.7
	Netherlands	11.9	0.0	29.3
	Switzerland	37.6	0.0	37.8
	Japan	28.3	22.8	45.9
*W* weights training	Austria	36.1	36.1	36.1
	Switzerland	29.3	29.3	29.3
	Japan	24.0	24.0	24.0
	Australia	10.7	10.7	10.7
C-V criterion	RMSPE	84.7	84.7	84.7

Notes: “ADH” stands for the results of Abadie et al. (2015), “Min.” and “Max.” denote minimal and maximal values, respectively, found under the condition that the corresponding predictor weights *V* lead to identical donor weights *W* in the training period. All numbers are given in percent, suppressing donors with weight less than 1%

15 Theoretically, it is possible that $W^{*}_{(\text {main})}(\widetilde V)$ and $W^{*}_{(\text {main})}(V^{*})$ coincide. However, that would be quite a coincidence.

16 One may prove that these conditions are not only necessary, but also sufficient for *W*^∗^ being a minimizer of Eq. (1).

17 It might be possible to strengthen the results of the rule of thumb to obtain a rigorous mathematical statement. This, however, is beyond the scope of this paper.

## References

[CR1] Abadie A, Diamond A, Hainmueller J (2010). Synthetic control methods for comparative case studies: estimating the effect of California’s tobacco control program. Journal of the American Statistical Association.

[CR2] Diamond A, Hainmueller J, Abadie, A (2011). Synth: an R package for synthetic control methods in comparative case studies. Journal of Statistical Software.

[CR3] Abadie A, Diamond A, Hainmueller J (2015). Comparative politics and the synthetic control method. American Journal of Political Science.

[CR4] Abadie A, Gardeazabal J (2003). The economic costs of conflict: a case study of the Basque country. The American Economic Review.

[CR5] Acemoglu D, Johnson S, Kermani A, Kwak J, Mitton T (2016). The value of connections in turbulent times: evidence from the United States. Journal of Financial Economics.

[CR6] Cavallo E, Galiani S, Noy I, Pantano J (2013). Catastrophic natural disasters and economic growth. The Review of Economics and Statistics.

[CR7] Gardeazabal J, Vega-Bayo A (2017). An empirical comparison between the synthetic control method and Hsiao et al.’s panel data approach to program evaluation. Journal of Applied Econometrics.

[CR8] Gobillon L, Magnac T (2016). Regional policy evaluation: interactive fixed effects and synthetic controls. The Review of Economics and Statistics.

[CR9] Kleven HJ, Landais C, Saez E (2013). Taxation and international migration of superstars: evidence from the European football market. The American Economic Review.

[CR10] R Core Team (2014). R: A Language and Environment for Statistical Computing.

